# Comparing stress and behavioral coping strategies during the early stages of the COVID-19 crisis among domestic and overseas Taiwanese

**DOI:** 10.1038/s41598-022-15567-y

**Published:** 2022-07-08

**Authors:** Cheng-Che Chen, Harry Yi-Jui Wu, Ming-Jui Yeh, Austin Horng-En Wang

**Affiliations:** 1 Department of Psychiatry, National Taiwan University Hospital Hsin-Chu Branch and Biomedical Park Hospital, Hsinchu, Taiwan; 2 Department of Psychiatry, College of Medicine, National Taiwan University, Taipei, Taiwan; 3 Cross College Elite Program, National Cheng Kung University, Tainan, Taiwan; 4 Institute of Health Policy and Management, College of Public Health, National Taiwan University, Taipei, Taiwan; 5 Department of Political Science, University of Nevada, Las Vegas, USA

**Keywords:** Diseases, Health care, Risk factors

## Abstract

This study reported domestic and overseas Taiwanese people’s perceived stress levels and examined the mediation effect of their coping strategies during the early stages of the COVID-19 pandemic. We recruited 2727 Taiwanese respondents from the COVIDiSTRESS Global Survey (N = 173,426) between March 30 and May 30, 2020. The self-report questionnaire included a modified 10-item Perceived Stress Scale and a 16-item coping strategy scale. Three stress-coping factors were extracted with principal component analysis and confirmatory factor analysis. Their effects were examined through a regression and mediation analysis. The overseas Taiwanese participants had a significantly higher stress level than domestic counterparts (2.89 to 2.69 in 1–5 scale, p < 0.001). Government guidance was associated with lower stress level among domestic (− 0.097, 95% C.I. [− 0.131, − 0.063]) but not overseas Taiwanese (0.025, [− 0.114, 0.163]). The association of stress level with residency was mediated by coping strategies, for government guidance (0.04, [0.01, 0.07], ref: domestic participants) and supportive social networks (− 0.03, [− 0.05, − 0.01]). All results hold after the propensity score matching on samples. Government guidance on COVID-19 as a channel for coping with stress is correlated with the residency status of the respondents. Public health authorities should recognize the importance of various mental health interventions during pandemics.

## Introduction

A new coronavirus (SARS-CoV-2) emerged as an acute respiratory syndrome (COVID-19) epidemic in humans and was centered in Wuhan, China in December 2019. The virus spread to hundreds of countries in a few months, and the World Health Organization (WHO) declared a global pandemic. Acute life-threatening stress through, e.g., the SARS disease outbreak in 2003, results in a sustained psychological impact^1^. However, the emergency preventative policies implemented by many governments and the WHO rely mainly on surveillance and early detection, community containment, and mass prophylaxis by using vaccines. The effectiveness of strategies for addressing mental and social aspects has been underestimated^2,3^.

The literature has shown that outbreaks of infectious diseases cause a broad spectrum of profound psychological effects in all people, not only in those with preexisting mental illnesses^4–6^. Psychological stress occurs when people perceive the environmental change that taxes their additional adaptive capacity to respond^7,8^. Stress related to the pandemic may be exacerbated by self-isolation policies that can increase social isolation and relationship difficulties globally^9^. During a pandemic, people experience feelings of helplessness, hopelessness, and fear induced by the threat of their own death and the death of family members and friends. Increasing media coverage and misinformation in early stages may induce anxiety in communities^10–12^. In particular, social and physical distancing strategies adopted in many countries during the COVID-19 pandemic have continuously affected the public’s mental health and have caused an economic recession^10,13,14^. Furthermore, stressors and negative emotions influence the immunity that interacts between the central nervous and endocrine systems to modify various antiviral responses^15^.

In the early days of the pandemic, studies of healthcare workers found that COVID-19 outbreaks were acute stressors^16^ and even related to acute stress disorder^17^; one study found similar effects of the pandemic crisis on mental health in the university^18^. Further, the study in Peru pointed out the relationship between anxiety, depression, and cognitive process strategies^19^, and people who were able to have positive relations with others and self-acceptance were less likely to have depression, anxiety, or stress reactions^20^. Those studies showed the importance of resilience and adaptive cognitive strategies between psychological distress and acute stress^21^. Meanwhile, some studies called mental health policy for protecting mental health during epidemics^22^, but the relationship between psychological stress, individual activities and social intervention is still unknown.

Therefore, the ways in which people cope with the stress caused by the COVID-19 pandemic warrant further investigation for social and behavioral science^9,23–25^. Some reports revealed the mental impact, trust, and worry during the pandemic in early 2020 in European and other countries^26–29^. Mental distress is associated with individual concern over disease, personal lockdown conditions, strictness of protective measures, and social support^29,30^. Methodologically speaking, the multiple sites analysis can identify the causal effect between the government’s policies and the citizens’ mental stress.

### Residency status, stress, and coping strategies

A pandemic may exacerbate the stigmatization of and discrimination against immigrants, which results in immigrants facing additional challenges when they experience acute stress and mental health problems while governments implement social isolation at the individual and community levels^31^. In the buffering hypothesis, social support may prevent stress appraisal for events or facilitate an adjustive counter response for illness prevention^32^. Immigrants who have emigrated from their country of origin may have less intergroup contact, access to formal assistance, social support, and cultural inclusion in their host countries^33,34^. Previous studies have shown that individuals’ demographic characteristics, stress types, and the community in which they live are related to their coping strategies^8,9,23,35^. There is a call to understand migrants’ psychological health and coping strategies during the ongoing COVID-19 pandemic, especially to understand the importance of government actions^23,36^.

The Taiwan Centers for Disease Control (Taiwan CDC) detected news about an outbreak with unknown etiology in Wuhan, China since January 2020^37,38^. Taiwan established real-time surveillance with rapid risk assessment, border control and quarantine, and laboratory capacity building in the early stage, and Taiwan achieved relatively successful control of the epidemic with an extremely low infection rate and mortality in the community^39–42^. The cumulative confirmed cases (case fatality rate) of COVID-19 were 442 (1.58%) in Taiwan, 16,751 (5.36%) in Japan, 183,410 (4.66%) in Germany, and 1.8 million (5.97%) in US on May 31-Jun 1, 2020^43^. In the worldwide web-based survey during March–May 2020, the proportions of current isolation status were 0.009% in Taiwan, 0.011% in Japan, 0.366% in Germany, and 0.597% in U.S., and Taiwan was the place of lowest isolated status over 200 countries^26,29^. The relatively low threat of COVID-19 makes the comparison between domestic and overseas Taiwanese a significant testbed to explore the issues related to citizens’ stress and coping strategies.

### Aims

Based on an analysis of data from the urgent online survey, we report the distributions and differences of the stress level and the coping strategies adopted by domestic and overseas Taiwanese individuals during the COVID-19 outbreak in early 2020. We further estimate the correlations of residency status, the stress level and coping strategies, and the mediation effect of coping strategies on the relationship between residency status and the stress level. By identifying the differences between domestic and overseas Taiwanese people, this study is the first of its kind to offer empirical evidence on the psychological health status and stress-coping behaviors of immigrants.

## Materials and methods

### Data collection

Taiwanese participants were recruited for participation in the COVIDiSTRESS global survey. This cross-cultural survey was designed to gain insight into psychological and behavioral responses while governmental orders such as staying home and canceling public functions were implemented in many countries during the early stages of the COVID-19 pandemic^26,29^. From March 30 to May 30, 2020, 173,426 respondents worldwide anonymously participated in the survey. The survey was translated into 47 languages via a dual translation procedure and was launched in 179 countries. The survey was preregistered on the Open Science Framework platform (https://osf.io/2ftma/) on March 30, 2020, and was approved by the Research Ethics Committee at Aarhus University, Denmark, with case number 2019-616-000009. The dataset is publicly available on the abovementioned website.

The respondents were invited by a Qualtrics link posted on the collaborators’ Facebook page, Twitter account, and private messaging app. All participants were invited to answer the survey titled “How is coronavirus affecting your life?” The survey has 113 questions. No reward was provided for completing this survey. All subjects received the consent form at the beginning of the survey, and they had to click the button to express their consent before taking the survey.

All participants were asked a series of sociodemographic questions in the first part. The participants were asked their primary language, age, gender, level of education, employment status, country of residence, and whether their country of residence is their home country. The survey did not ask the respondents to indicate their biological sex.

### Domestic and overseas Taiwanese participants

We identified domestic and overseas Taiwanese participants according to the following procedure. Domestic Taiwanese were defined as those who lived in Taiwan, answered the traditional Chinese version (ZH-T), and considered Taiwan to be their home country. Overseas Taiwanese individuals were defined as those who did not live in Taiwan, answered the ZH-T version, and did not consider their current residence as their home country. Among all 3089 ZH-T respondents, 2469 (79.9%) were defined as domestic Taiwanese, 258 (8.3%) were defined as overseas Taiwanese, and 362 (11.7%) who did not fit the criteria mentioned above were excluded from further analysis. We equated the respondents who selected the traditional Chinese version in Taiwan and abroad as Taiwanese for three practical reasons. First, this survey was not disseminated in Hong Kong, which is another major region that uses traditional Chinese. Second, most overseas Chinese—including those in Singapore, Malaysia, Indonesia, and the U.S.—were offered the simplified Chinese version at the beginning of the survey. Third, we used the item “considering their current residency as the home country” to further screen possible overseas Taiwanese, which may mitigate the issue of second-generation overseas Chinese or Taiwanese and exclude foreigners in Taiwan. While different contextual factors would influence the overseas participants living in different countries, they have certain common features that mark them off from the domestic participants, prominently, their immigration status and other factors that are tied to this status. This common feature makes the overseas comparable when estimating the mediation effects of coping strategies.

### Level of stress

All participants were asked to complete a modified version of the 10-item Perceived Stress Scale (PSS-10)^44–46^. The participants were asked to evaluate their condition “in the last week” instead of “last month”, and this modification was made to capture the rapidly changing situation during the pandemic^26,29^. As measured by the Cronbach’s alpha, the internal consistency of the scale ranged from 0.66 to 0.90^29^. In our selected data, the Cronbach’s alpha for the ten items answered by the Taiwanese participants was 0.898. We therefore averaged the responses to create a single indicator of the participants’ stress level when the participants were answering the question (range: 1–5, mean: 2.70, variance: 0.54).

### Coping strategies

In the second part, the participants were asked sets of items related to people’s experiences of distress and worry during the COVID-19 epidemic and items of coping behaviors^26,29^. The items of coping behaviors were depicted in the section title—“I have found the following helpful for coping with feelings of discomfort raised by the Coronavirus situation”: Q1—“Information from the government”; Q2—“Face-to-face interactions with friends and family”; Q3—“Phone calls or other long-range interactions with friends and family”; Q4—“Face-to-face interactions with colleagues”; Q5—“Phone calls or other long-range interactions with colleagues”; Q6—“Social media”; Q7—“Video games (alone)”; Q8—“Video games (online)”; Q9—“Watching T.V. shows or movies”; Q10—“Dedicating myself to helping others”; Q11—“Dedicating myself to preparing for the crisis”; Q12—“Dedicating myself to following the government’s advice”; Q13—“Dedicating myself to my work/vocation”; Q14—“Dedicating myself to an activity or hobby”; Q15—“God, religion or spirituality”; and Q16—“Knowledge of actions taken by the government or civil services”. The Cronbach’s alpha for the sixteen items answered by the Taiwanese participants was 0.831.

### Data analyses

We compared the demographic backgrounds, level of stress, and the reliance of coping strategies between domestic and overseas Taiwanese respondents through the two-group T-test and Chi-squared test. A principal component analysis with the varimax rotation method was applied to reduce the 16 coping strategies to make the results more interpretable^47^. All three factors with eigenvalues larger than 1 were the independent variables, while the government action-related factor served as the item of interest^48^.

Estimating the effect of the country of residence raises the potential threat that differences in the individual sociodemographic backgrounds of the domestic and overseas Taiwanese participants may be associated with their level of stress. A linear regression analysis was used to estimate the effect of the government’s response via the Taiwanese dataset (n = 2727). The dependent variable was the level of stress. The control variables included a dummy variable for the country of residence, age, gender, education, and employment. A mediation analysis was further applied to examine whether the coping strategies mediated the effect of residency on their level of stress^49^. The analysis was conducted by using the Psych package in R 3.1.3. All three major factors were set as mediators, while residency and stress level were independent and dependent variables.

We also applied a case–control design to reduce the potential threat to compare stress levels for triangulation (supplement)^50,51^. Propensity score matching was used to select the domestic participants who shared a similar background with the overseas participants^51,52^. The matching was conducted by using the MatchIt package in R 3.1.3 with the *nearest* method.

### Use of human participants

The survey was preregistered on the Open Science Framework platform (https://osf.io/2ftma/) on March 30, 2020, and was approved by the Research Ethics Committee at Aarhus University, Denmark, with case number 2019-616-000009. The dataset is publicly available on the abovementioned website. All identifiable information of respondents were removed before the data was released and analysed. All research was performed in accordance with relevant guidelines/regulations. Informed consent of all respondents were obtained when the survey was conducted.

## Results

### Demographic characteristics and the level of stress

The characteristics and stress levels of the respondents are shown in Table 1 (n = 2727). The mean ages of domestic and overseas participants were 32.8 and 33.1 years (p = 0.6). More women than men responded in both domestic and overseas groups (70.5% and 64.3%, p = 0.21). After matching, there were no significant differences between the overseas and domestic Taiwanese participants in terms of education (p = 0.76) and employment status (p = 0.94) (Table S2, n = 396). The distributions of the variables after the propensity score matching analysis can be found in Table S2. Among the 258 overseas respondents, 98 (38.0%) lived in the United States, 50 (19.4%) lived in Japan, 14 (5.4%) lived in Netherlands, 13 (5.0%) lived in Germany, and 73 (28.3%) lived in other countries.

**Table 1:** Sociodemographic factors of the Taiwanese participants (n = 2727).

	Domestic (n = 2469)	Overseas (n = 258)	Test	*p*-value
**Age (years)**			T-test	0.64
Mean (S.D.)	32.8 (11.2)	33.1 (9.6)		
Min	18	18		
Max	82	100		
**Gender**			χ^2^	0.21
Male (%)	664 (26.9%)	85 (32.9%)		
Female (%)	1742 (70.5%)	166 (64.3%)		
Other (%)	62 (2.5%)	7 (2.7%)		
**Education**			χ^2^	< 0.001
Senior High and below	209 (8.2%)	12 (4.7%)		
College	1532 (62.1%)	93 (36.0%)		
Graduate	725 (29.3%)	153 (59.3%)		
**Employment**			χ^2^	< 0.001
Students	556 (22.5%)	81 (31.7%)		
Full-time	1343 (54.5%)	114 (44.7%)		
Part-time	115 (4.6%)	21 (8.2%)		
Self-employed	190 (7.7%)	6 (2.3%)		
Unemployed	185 (7.5%)	25 (9.8%)		
Retired	74 (3.0%)	8 (3.1%)		
PSS-10 score (SD)	2.69 (0.73)	2.89 (0.80)	T-test	< 0.001

^a^χ^2^: Chi-squared test.

There was a significant difference in the perceived level of stress on the PSS-10 between the overseas and domestic Taiwanese participants before matching (2.89 to 2.69, p < 0.001), and the difference was also significant after matching (2.89 to 2.61, p < 0.001) (Table S2).

### Differences in the coping strategies among participants

The coping strategies that the domestic and overseas Taiwanese participants used are shown in Table 2 (n = 2727). Among the domestic Taiwanese participants, the top three strategies used to reduce stress were “Knowledge of actions taken by the government or civil services (Q16, 4.93)”, “Information from the government (Q1, 4.81)”, and “Dedicating myself to an activity or hobby (Q14, 4.81)”. Among the overseas Taiwanese participants, the top three strategies were “Dedicating myself to an activity or hobby (Q14, 4.73)”, “Phone calls or other long-range interactions with friends and family (Q3, 4.58)”, and “Watching T.V. shows or movies (Q9, 4.49)”. Given the similar sociodemographic backgrounds after propensity score matching, there was still significant difference in the coping strategies chosen by the domestic and overseas Taiwanese participants. The coping strategy scores of the matched participants are shown in Table S3. The coping strategies of Q1, Q12, and Q16 are government-related actions or information, and these items’ scores are higher among domestic Taiwanese than the overseas Taiwanese in both unmatched and matched comparisons (Table 2, Table S3). The scores of Q4, Q7, Q8, and Q11 are higher among the domestic than the overseas Taiwanese. The score of Q5 “Phone calls or other long-range interactions with colleagues” is relatively high among overseas Taiwanese, but the difference is insignificant after matching.

**Table 2:** Coping strategies of the Taiwanese participants (n = 2727).

Coping strategies	Domestic (n = 2469)	Overseas (n = 258)	Diff. (T-test)
Q1. Information from the government	**4.81**^a^	3.94	p < 0.001
Q2. Face-to-face interactions with friends and family	4.16	4.01	p = 0.10
Q3. Phone calls or other long-range interactions with friends and family	4.45	4.58	p = 0.09
Q4. Face-to-face interactions with colleagues	**3.41**	3.16	p = 0.01
Q5. Phone calls or other long-range interactions with colleagues	3.81	**4.05**	p = 0.007
Q6. Social media	4.12	4.07	p = 0.59
Q7. Video games (alone)	**4.01**	3.65	p = 0.001
Q8. Video games (online)	**3.74**	3.46	p = 0.02
Q9. Watching T.V. shows or movies	4.44	4.49	p = 0.60
Q10. Dedicating myself to helping others	4.10	4.07	p = 0.80
Q11. Dedicating myself to preparing for the crisis	**4.26**	4.08	p = 0.03
Q12. Dedicating myself to following the government’s advice	**4.68**	4.05	p < 0.001
Q13. Dedicating myself to my work/vocation	4.12	4.03	p = 0.38
Q14. Dedicating myself to an activity or hobby	4.81	4.73	p = 0.31
Q15. God, religion or spirituality	2.83	2.72	p = 0.33
Q16. Knowledge of actions taken by the government or civil services	**4.93**	4.28	p < 0.001

^a^Each item scoring is 1–6: 1—strongly disagree, 6—strongly agree.

Significant values are in bold.

The principal component analysis with a varimax rotation^51^ reveals that among the 16 coping strategies from all domestic and overseas Taiwanese respondents (Table 3, n = 2727), three optimal components with eigenvalues larger than 1 explain 53% of the overall variance. All three government-related coping strategies (Q1, Q12, and Q16) are heavily loaded on the first component, while the other strategies are lower than 0.6 (all loadings larger than 0.6 are marked in Table 3). The second component is mainly about supportive social networks (Q2, Q4, Q5, Q10), such as interactions with colleagues and friends; the third component includes mostly strategies that involve personal entertainment (Q7, Q8), such as video games and television. The loadings can be found in Table 3.

**Table 3:** Principal component analysis of coping strategies (with a varimax rotation) (n = 2727).

	Factor 1 (government guiding)	Factor 2 (supportive social networks)	Factor 3 (personal entertainment)
**Q16. Knowledge of actions taken by the government or civil services**	**0.87**	0.05	0.11
**Q1. Information from the government**	**0.85**	0.05	0.08
**Q12. Dedicating myself to following the government's advice**	**0.82**	0.15	0.12
Q11. Dedicating myself to preparing for the crisis	0.56	0.33	0.12
Q14. Dedicating myself to an activity or hobby	0.49	0.12	0.48
Q6. Social media	0.38	0.31	0.21
**Q4. Face-to-face interactions with colleagues**	− 0.07	**0.74**	0.20
**Q5. Phone calls or other long-range interactions with colleagues**	0.16	**0.71**	0.07
**Q10. Dedicating myself to helping others**	0.34	**0.61**	0.04
**Q2. Face-to-face interactions with friends and family**	0.20	**0.60**	0.03
Q15. God, religion or spirituality	− 0.05	0.57	− 0.03
Q13. Dedicating myself to my work/vocation	0.23	0.55	0.05
Q3. Phone calls or other long-range interactions with friends and family	0.44	0.49	0.20
**Q7. Video games (alone)**	0.09	− 0.10	**0.88**
**Q8. Video games (online)**	0.04	0.02	**0.87**
Q9. Watching T.V. shows or movies	0.30	0.17	0.48
Sum of squares loadings	3.38	2.92	2.14
Variance explained	21%	18%	13%
Overall variance explained	53%		

Significant values are in bold.

A confirmatory factor analysis (CFA) was further carried out for the three main factors generated in Table 3. When the bold factors in Table 3 were assigned to the three factors, the overall comparative fit index (CFI) was 0.949 with a root mean square error of approximation (RMSEA) of 0.082 (95% confidence interval (C.I.) [0.074–0.089]), and all nine items had a loading larger than 0.6 on the three factors. When all factors in Table 3 were assigned to the three factors according to their heaviest loaded factor, the CFA shows that the CFI 0.831 had a RMSEA of 0.095 (95% C.I. [0.091–0.099]), while all 16 items except for two had a loading larger than 0.6. Since the coping strategies asked in the survey were not meant to establish a clear theory-driven construct, it is not unreasonable that the three factors only provide a moderate fit to the 16 coping strategies. Nevertheless, the three factors can still be used to summarize the coping strategies categories and estimate the relationship between the demographic factors and the stress levels.

### The coping strategies and the mediation effect of residency

As summarized in Table 4, the regression analysis showed a significant correlation between government-related coping strategies and the level of stress among the domestic Taiwanese but not among the overseas Taiwanese participants. Models 1 and 2 were used to explain the level of stress among the domestic Taiwanese participants, while Models 3 and 4 were used for the overseas Taiwanese participants. Models 1 and 3 included only the three factors of coping strategies, while Models 2 and 4 also included the respondents’ sociodemographic characteristics (age, gender, education, and employment status). After controlling for other sociodemographic characteristics, the partial coefficient of the first component, in which government guidance is heavily loaded, was still significantly negative for the domestic Taiwanese participants in Models 1 and 2 (− 0.097, p < 0.001, 95% C.I. [− 0.131, − 0.063]) but not the overseas Taiwanese participants in Models 3 and 4 (0.010, p = 0.72, 95% C.I. [− 0.126, 0.145]).

**Table 4:** Coping strategies and the level of stress (n = 2727).

	Model 1^c^ Domestic (n = 2469)	Model 2^c^ Domestic—full (n = 2469)	Model 3^c^ Overseas (n = 258)	Model 4^c^ Overseas—full (n = 258)
**Factor 1 (government guidance)**	− 0.081*** [− **0.116,** − **0.047]**	− 0.097*** [− **0.131,** − **0.063]**	**0.001 [**− **0.124, 0.127]**	**0.010 [**− **0.126, 0.145]**
Factor 2 (supportive social networks)	− 0.136*** [− 0.171, − 0.101]	− 0.090*** [− 0.125, − 0.055]	− 0.253*** [− 0.374, − 0.131]	− 0.227*** [− 0.365, − 0.090]
Factor 3 (personal entertainment)	0.084*** [0.049, 0.119]	0.026 [− 0.011, 0.63]	− 0.137** [− 0.257, − 0.018]	− 0.152** [− 0.280, − 0.024]
Age^b^		Yes		Yes
Gender^b^		Yes		Yes
Education^b^		Yes		Yes
Employment^b^		Yes		Yes
Constant	2.720*** [2.685, 2.755]	3.202*** [3.041, 3.363]	2.651*** [2.530, 2.773]	3.044*** [2.366, 3.723]
Adjusted R^2^	0.059	0.145	0.160	0.237
Variance inflation factor	1.00	2.29	1.01	3.55

^a^*p < 0.05, **p < 0.01, ***p < 0.001.

^b^The coefficients of the control variables are neglected because the variables were coded as a long list of dummies. The complete result can be provided upon request.

^c^Models 1 and 2 are used to explain the level of stress among the domestic Taiwanese participants, and Model 2 includes the respondents’ sociodemographic characteristics: age, gender, education, and employment status; Models 3 and 4 are used to explain the level of stress among the overseas Taiwanese participants, and Model 4 also includes the respondents’ sociodemographic characteristics.

Significant values are in bold.

Meanwhile, the second component (which is mostly related to the supportive social networks) is associated with the participants’ level of stress for both groups of participants. The individual coping strategy (factor 3, which is mostly about playing video games and watching T.V.) is associated with the level of stress among overseas but not among domestic Taiwanese.

**Figure 1:**
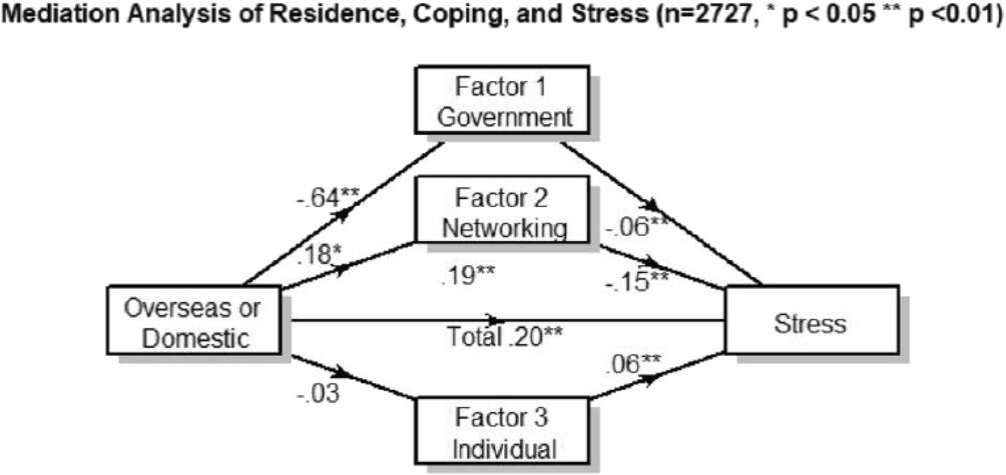
Mediation analysis of the country of residence, coping, and stress (n = 2727).

The vanishing significance of the first factor in Models 3 and 4 in Table 4 suggests the possible mediation effect of the coping strategies. The results of the mediation analysis are shown in Fig. 1. Mediation analysis identifies the direct association of the overseas residency with the level of stress (0.20, p < 0.01); after adding the three coping strategy factors, the direct effect of residence is still significant (0.19, p < 0.001). Meanwhile, bootstrapping shows that the mediation effect of government guidance is associated with stress level (0.04, 95% C.I. [0.01, 0.07]) among overseas participants, and the effect of supportive social networks (− 0.03, 95% C.I. [− 0.05, − 0.01]) is statistically significant for diminishing stress level, while personal entertainment (factor 3) is not (0.00, 95% C.I. [− 0.01, 0.01]). The results are relevant to the logistic regression.

## Discussion

The study shows that the overseas Taiwanese participants experienced a higher level of stress than the domestic Taiwanese participants during the early stages of the COVID-19 crisis, even after controlling for sociodemographic characteristics. The psychological stress of the pandemic on individuals was associated with residency and also mediated by coping strategies: government’s action and information provision, personal social networks, and entertainment. While further research is needed to estimate the effects of residency status on stress-coping behaviors under pandemics, our findings have indicated the differences in coping strategies between domestic and overseas Taiwanese. The early actions taken by the Taiwanese government may help domestic respondents, but those overseas participants did not benefit from government guidance. This difference might be explained by the residency status of these overseas Taiwanese as previous studies have shown that immigrants or people involved in diaspora face additional challenges^33,34^.

First, the analyses found that the domestic Taiwanese participants tended to trust the government. As people have more power and the right to hold the government accountable, they express more faith in governmental decisions regarding disease control and prevention^41^. Taiwan ensured the coordination of different government agencies and activated the Central Epidemic Command Center (CECC) with daily reassurance and education for the public beginning in January 2020^38–41^. To improve the public’s awareness of prevention, the CECC provided daily updates on surveillance, the number of confirmed cases, and infection control information (including face mask management, physical distancing principles, and guides for screening and quarantine via public press conferences, Facebook, and Line). These procedures provided accurate information and enhanced the social support system to help people maintain a normal lifestyle. Not all exposure to media has the same effect. If media reports focus on what to do instead of the crisis itself, then they can reduce stress, as shown in our study^41,53^. Thus, it is also reasonable that the domestic Taiwanese participants would agree that they engage in activities based on the government’s advice to prepare for the crisis.

Second, to the contrary, the overseas Taiwanese participants, who are immigrants, may not enjoy the same level of political power and rights in their host countries and lack other forms of social participation and cultural inclusion. The strategies of these participants tend to be more inward-focused, such as personal activities, hobbies, and interactions with friends and family. Regarding using these two strategies, there were no significant differences between the overseas and domestic Taiwanese participants.

Compared to the overseas Taiwanese participants, the domestic Taiwanese participants reported significantly higher levels of agreement with the use of coping strategies. This finding might be associated with the lower stress level of the domestic Taiwanese participants, which implies that the strategies that they adopted enabled them to cope more successfully with stress. However, considering the relatively successful containment of the COVID-19 epidemic in Taiwan, the differences in the level of stress might simply be due to the different experiences of the intensity of the epidemic. Furthermore, the current study falls short of exploring the resilience developed among immigrants to help them adapt well in the face of various adversities.

### Limitations

This study has several limitations. First, the number of overseas respondents was too small to analyze the connection between their perceived level of stress and the governmental responses in their various countries of residence. We constructed the overseas Taiwanese as a meaningful comparison group in the sense that Taiwan’s relatively successful control of the pandemic in March and April of 2020 (more than 60% of the overseas Taiwanese respondents lived in North America, the Netherlands, and Germany). This limitation may not have biased our main findings.

Second, perceived stress may be influenced by experiences of infected family members, personality, self-efficacy, cognitive coping strategies or preexisting psychiatric disorders, but the COVIDiSTRESS dataset did not include such items^17,19,21,26^. We controlled the sociodemographic factors to estimate the “treatment” effect of the country of residence. Nevertheless, these aspects should be considered in further investigations of the relationship between stress and government responses.

Third, there is still no consensus in the literature on the definition of stress. The PSS-10 used in this study is widely accepted as a reliable measure of stress, and PSS-10 scores are highly correlated with anxiety and depression^1,54^. The connection between short-term stress coping strategies and long-term mental health still needs further investigation^4,24,55^.

Fourth, the data collection process of the emergency mental health survey cannot fully represent the demographics of Taiwanese people^56^. Generally, relatively young individuals and females were overrepresented in the unmatching dataset^26,29^. Given the various sociodemographic backgrounds of the overseas respondents, we applied the propensity score matching method to match the domestic respondents with similar backgrounds. The matched samples are still by no mean representative. Hence, the result may still suffer from the issue of generalizability.

Fifth, since the coping strategies asked in the survey were not meant to build a clear theory-driven construct, it is not unreasonable that the three factors only provided a moderate fit to the 16 coping strategies. Nevertheless, the three factors can still be used to summarize coping strategies and estimate the relationship between the factors and the stress level. While there might well be other explanatory factors for the differences in level of stress between domestic and overseas Taiwanese, the preliminary findings of the significant correlations between complying with government policies and stress relief among the respondents offer valuable insight that government intervention might be important for mental health during the early stages of the COVID-19 outbreak.

## Conclusion

This study finds that, compared with the overseas Taiwanese respondents, those domestic ones had a lower level of stress and might rely on government-related coping strategies during the early stages of the COVID-19 pandemic. The correlation between the respondents’ residency status and their perceived level of stress is mediated by the three types of coping strategies, including government guidance and individual support. Our results provide evidence that the perceived stress among a population is related to the government’s immediate response, thereby suggesting the connection between the government and community resilience. Dealing with stress during pandemics is a substantial and dynamic challenge worldwide. Prolonged states of emergency and stressors related to isolation could decrease compliance with set behavioral objectives during pandemics, especially among immigrants^10,29^. When facing the formidability of COVID-19, it is useful for researchers to go back to pre-Hans Selye’s time before the science of stress became the investigation of the neurohormonal regulation of damage/defense reactions^57^. Admittedly, the definition of stress has evolved with immense influence by political economies and sociocultural realities. Nevertheless, this “new normal” forming in the future might fundamentally transform the world's definition of productivity and rewrite the social contract in demand of care and government accountability. Hence we suggest that intense collaboration and communication is needed among governments, scientists, and healthcare professionals.

## Supplementary Information

Supplementary Information.

## Data Availability

The data used in this article is publicly available at Ref. ^29^.

## References

[1] McAlonan GM, Lee AM, Cheung V, Cheung C, Tsang KW, Sham PC (2007). Immediate and sustained psychological impact of an emerging infectious disease outbreak on health care workers. Can. J. Psychiatry.

[2] Holmes EA, O'Connor RC, Perry VH, Tracey I, Wessely S, Arseneault L (2020). Multidisciplinary research priorities for the COVID-19 pandemic: A call for action for mental health science. Lancet Psychiatry.

[3] Reissman DB, Watson PJ, Klomp RW, Tanielian TL, Prior SD (2006). Pandemic influenza preparedness: Adaptive responses to an evolving challenge. J. Homeland Secur. Emerg. Manage..

[4] Wang Y, Di Y, Ye J, Wei W (2021). Study on the public psychological states and its related factors during the outbreak of coronavirus disease 2019 (COVID-19) in some regions of China. Psychol. Health Med..

[5] World Health Organization. *Mental Health and Psychosocial Considerations During the COVID-19 Outbreak*. World Health Organization (2020). https://www.who.int/publications-detail/mental-health-and-psychosocial-considerations-during-the-covid-19-outbreak. Accessed 16 May 2020.

[6] World Health Organization, Regional Office for the Western Pacific. *Mental Health and Psychosocial Support Aspects of the COVID-19 Response Manila: Regional Office for the Western Pacific* (2020). https://apps.who.int/iris/handle/10665/331927. Accessed 22 May 2020.

[7] Chrousos GP, Gold PW (1992). The concepts of stress and stress system disorders. Overview of physical and behavioral homeostasis. JAMA.

[8] Cohen S, Rodriquez MS (1995). Pathways linking affective disturbances and physical disorders. Health Psychol..

[9] Bavel JJV, Baicker K, Boggio PS, Capraro V, Cichocka A, Cikara M (2020). Using social and behavioural science to support COVID-19 pandemic response. Nat. Hum. Behav..

[10] Brooks SK, Webster RK, Smith LE, Woodland L, Wessely S, Greenberg N (2020). The psychological impact of quarantine and how to reduce it: Rapid review of the evidence. The Lancet.

[11] Kar SK, Yasir Arafat SM, Kabir R, Sharma P, Saxena SK, Saxena SK (2020). Coping with mental health challenges during COVID-19. Coronavirus Disease 2019 (COVID-19). Medical Virology: From Pathogenesis to Disease Control.

[12] Lima CKT, Carvalho PMM, Lima I, Nunes J, Saraiva JS, de Souza RI (2020). The emotional impact of Coronavirus 2019-nCoV (new Coronavirus disease). Psychiatry Res..

[13] McKee M, Stuckler D (2020). If the world fails to protect the economy, COVID-19 will damage health not just now but also in the future. Nat. Med..

[14] Middaugh JP (2008). Pandemic influenza preparedness and community resiliency. JAMA.

[15] Glaser R, Kiecolt-Glaser JK (2005). Stress-induced immune dysfunction: Implications for health. Nat. Rev. Immunol..

[16] Wu W, Zhang Y, Wang P, Zhang L, Wang G, Lei G (2020). Psychological stress of medical staffs during outbreak of COVID-19 and adjustment strategy. J. Med. Virol..

[17] Shahrour G, Dardas LA (2020). Acute stress disorder, coping self-efficacy and subsequent psychological distress among nurses amid COVID-19. J. Nurs. Manage..

[18] Amaral-Prado HM, Borghi F, Mello T, Grassi-Kassisse DM (2021). The impact of confinement in the psychosocial behaviour due COVID-19 among members of a Brazilian university. Int. J. Soc. Psychiatry.

[19] Ames-Guerrero RJ, Barreda-Parra VA, Huamani-Cahua JC, Banaszak-Holl J (2021). Self-reported psychological problems and coping strategies: A web-based study in Peruvian population during COVID-19 pandemic. BMC Psychiatry.

[20] Lopes AR, Nihei OK (2021). Depression, anxiety and stress symptoms in Brazilian university students during the COVID-19 pandemic: Predictors and association with life satisfaction, psychological well-being and coping strategies. PLoS ONE.

[21] Ye Z, Yang X, Zeng C, Wang Y, Shen Z, Li X (2020). Resilience, social support, and coping as mediators between COVID-19-related stressful experiences and acute stress disorder among college students in China. Appl. Psychol. Health Well Being.

[22] Lestari R, Setyawan FEB (2021). Mental health policy: Protecting community mental health during the COVID-19 pandemic. J. Public Health Res..

[23] Centers for Disease Control and Prevention. *Coping with Stress: Mental Health and Coping During COVID-19 2021* (2021). https://www.cdc.gov/coronavirus/2019-ncov/daily-life-coping/managing-stress-anxiety.html. Accessed 22 Jan 2020.

[24] Ransing R, Ramalho R, Orsolini L, Adiukwu F, Gonzalez-Diaz JM, Larnaout A (2020). Can COVID-19 related mental health issues be measured?. Brain Behav. Immun..

[25] Szczesniak D, Ciulkowicz M, Maciaszek J, Misiak B, Luc D, Wieczorek T (2020). Psychopathological responses and face mask restrictions during the COVID-19 outbreak: Results from a nationwide survey. Brain Behav. Immun..

[26] Lieberoth A, Lin SY, Stockli S, Han H, Kowal M, Gelpi R (2021). Stress and worry in the 2020 coronavirus pandemic: Relationships to trust and compliance with preventive measures across 48 countries in the COVIDiSTRESS global survey. R. Soc. Open Sci..

[27] Petersen MW, Dantoft TM, Jensen JS, Pedersen HF, Frostholm L, Benros ME (2021). The impact of the Covid-19 pandemic on mental and physical health in Denmark—A longitudinal population-based study before and during the first wave. BMC Public Health.

[28] Helsingen LM, Refsum E, Gjostein DK, Loberg M, Bretthauer M, Kalager M (2020). The COVID-19 pandemic in Norway and Sweden—threats, trust, and impact on daily life: A comparative survey. BMC Public Health.

[29] Yamada Y, Cepulic DB, Coll-Martin T, Debove S, Gautreau G, Han H (2021). COVIDiSTRESS global survey dataset on psychological and behavioural consequences of the COVID-19 outbreak. Sci. Data.

[30] Rachev NR, Han H, Lacko D, Gelpi R, Yamada Y, Lieberoth A (2021). Replicating the disease framing problem during the 2020 COVID-19 pandemic: A study of stress, worry, trust, and choice under risk. PLoS ONE.

[31] World Health Organization. *Social Stigma Associated with COVID-19—A Guide to Preventing and Addressing Social Stigma 2020*. https://www.who.int/docs/default-source/coronaviruse/covid19-stigma-guide.pdf. Accessed 2 June 2020.

[32] Cohen S, Wills TA (1985). Stress, social support, and the buffering hypothesis. Psychol. Bull..

[33] Lazarus RS (1985). The psychology of stress and coping. Issues Ment Health Nurs..

[34] Lee RM, Noh CY, Yoo HC, Doh HS (2007). The psychology of diaspora experiences: Intergroup contact, perceived discrimination, and the ethnic identity of Koreans in China. Cult. Divers. Ethnic Minor. Psychol..

[35] Garfin DR, Thompson RR, Holman EA (2018). Acute stress and subsequent health outcomes: A systematic review. J. Psychosom. Res..

[36] Chander R, Murugesan M, Ritish D, Damodharan D, Arunachalam V, Parthasarathy R (2020). Addressing the mental health concerns of migrant workers during the COVID-19 pandemic: An experiential account. Int. J. Soc. Psychiatry.

[37] Yeh MJ, Cheng Y (2020). Policies tackling the COVID-19 pandemic: A sociopolitical perspective from Taiwan. Health Secur..

[38] Cheng HY, Li SY, Yang CH (2020). Initial rapid and proactive response for the COVID-19 outbreak—Taiwan's experience. J. Formos Med. Assoc..

[39] Lin C, Braund WE, Auerbach J, Chou JH, Teng JH, Tu P (2020). Policy decisions and use of information technology to fight COVID-19, Taiwan. Emerg. Infect. Dis..

[40] Lo HA, Huang JJ, Chen CC, Tsai D, Chou FH, Shieh V (2020). Community-based epidemic prevention in Taiwan: Combating the coronavirus disease-2019 crisis. Disaster Med. Public Health Prep..

[41] Wang CJ, Ng CY, Brook RH (2020). Response to COVID-19 in Taiwan: Big data analytics, new technology, and proactive testing. JAMA.

[42] Ng TC, Cheng HY, Chang HH, Liu CC, Yang CC, Jian SW (2021). Comparison of estimated effectiveness of case-based and population-based interventions on COVID-19 containment in Taiwan. JAMA Intern. Med..

[43] Coronavirus Pandemic (COVID-19) (2020). https://ourworldindata.org/coronavirus. Accessed 8 Oct 2021.

[44] Chiu YH, Lu FJ, Lin JH, Nien CL, Hsu YW, Liu HY (2016). Psychometric properties of the perceived stress scale (PSS): Measurement invariance between athletes and non-athletes and construct validity. PeerJ.

[45] Leung DY, Lam TH, Chan SS (2010). Three versions of perceived stress scale: Validation in a sample of Chinese cardiac patients who smoke. BMC Public Health.

[46] Ng SM (2013). Validation of the 10-item Chinese perceived stress scale in elderly service workers: One-factor versus two-factor structure. BMC Psychol..

[47] Hammig O (2019). Health risks associated with social isolation in general and in young, middle and old age. PLoS ONE.

[48] Hayton JC, Allen DG, Scarpello V (2004). Factor retention decisions in exploratory factor analysis: A tutorial on parallel analysis. Organ. Res. Methods.

[49] Baron RM, Kenny DA (1986). The moderator-mediator variable distinction in social psychological research: Conceptual, strategic, and statistical considerations. J. Pers. Soc. Psychol..

[50] Cannon M, Jones P, Huttunen MO, Tanskanen A, Huttunen T, Rabe-Hesketh S (1999). School performance in Finnish children and later development of schizophrenia: A population-based longitudinal study. Arch. Gen. Psychiatry.

[51] Walsh MC, Trentham-Dietz A, Newcomb PA, Gangnon R, Palta M (2012). Using propensity scores to reduce case-control selection bias. Epidemiology.

[52] Rosenbaum PR, Rubin DB (1984). Reducing bias in observational studies using subclassification on the propensity score. J. Am. Stat. Assoc..

[53] Garfin DR, Silver RC, Holman EA (2020). The novel coronavirus (COVID-2019) outbreak: Amplification of public health consequences by media exposure. Health Psychol..

[54] Lu W, Bian Q, Wang W, Wu X, Wang Z, Zhao M (2017). Chinese version of the perceived stress scale-10: A psychometric study in Chinese university students. PLoS ONE.

[55] Kubzansky LD, Winning A, Kawachi I (2014). Affective States and Health. Social Epidemiology.

[56] Pierce M, McManus S, Jessop C, John A, Hotopf M, Ford T (2020). Says who? The significance of sampling in mental health surveys during COVID-19. Lancet Psychiatry.

[57] Jackson M (2013). The Age of Stress: Science and the Search for Stability.

