# Supplementary material for: Comparing stress and behavioral coping strategies during the early stages of the COVID-19 crisis among domestic and overseas Taiwanese
Source: Sci Rep. 2022 Jul 8;12:11613. doi: 10.1038/s41598-022-15567-y (PMC9264308; doi:10.1038/s41598-022-15567-y)
Supplement: Supplementary file 1 — Supplementary Information. [file 41598_2022_15567_MOESM1_ESM.docx]

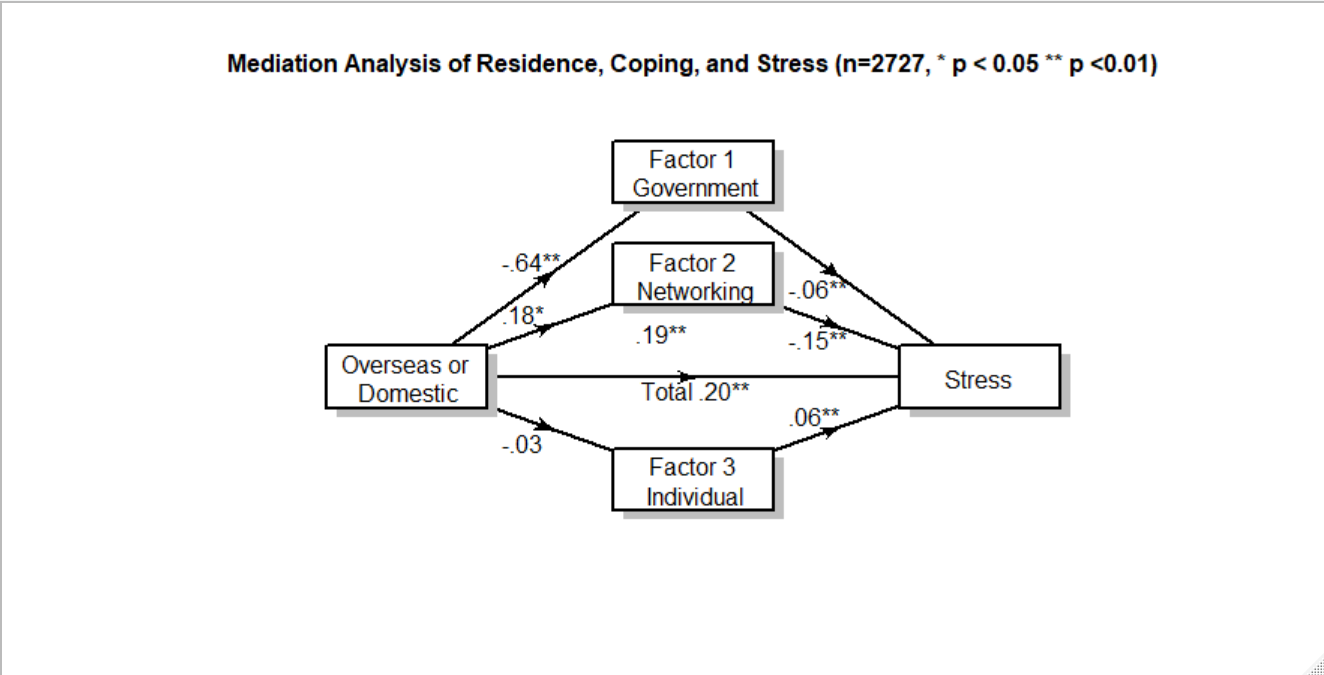


**Fig S1 Mediation Analysis of Residence, Coping, and Stress**

^a^ **p* <0.05, ***p*<0.01

^b^ Residential status: Overseas (ref: domestic status)

^c^ Direct effect with stress level: 0.2

^d^ Effect: Overseas🡪 Factor 1 (government guidance) 🡪 stress level: 0.04; Overseas 🡪 Factor 2 (supportive social networks) 🡪 stress level: -0.03; Overseas 🡪 Factor 3 (personal entertainment) 🡪 stress level: -0.0018

Supplementary information

**S1 Table.** Sociodemographic Factors of the matched Taiwanese Participants (n=386)

|  | | Domestic  (n=193) | Overseas  (n=193) | Test | *p*-value |
| --- | --- | --- | --- | --- | --- |
| Age (years) | |  |  | T-test, t = -0.0679 | 0.473 |
| Mean (S.D.) | | 33.3 (9.13) | 32.7 (7.91) |  |  |
| Min | | 19 | 18 |  |  |
| Max | | 64 | 64 |  |  |
| Gender | |  |  | χ^2^ = 0.22 | 0.89 |
| Male (%) | | 59 (30.6%) | 60 (31.1%) |  |  |
| Female (%) | | 132 (68.4%) | 130 (67.4%) |  |  |
| Other (%) | | 2 (1.0%) | 3 (1.5%) |  |  |
| Education | |  |  | χ^2^ = 1.15 | 0.77 |
| Senior High and below | | 5 (2.6%) | 6 (3.1%) |  |  |
| College | | 64 (33.2%) | 64 (33.2%) |  |  |
| Graduate | | 124 (64.2%) | 123 (63.7%) |  |  |
| Employment | |  |  | χ^2^ = 1.23 | 0.94 |
| Students | | 52 (26.9%) | 57 (29.5%) |  |  |
| Full-time | | 95 (49.2%) | 89 (46.1%) |  |  |
| Part-time | | 18 (9.3%) | 17 (8.8%) |  |  |
| Self-employed | | 6 (3.1%) | 5 (2.6%) |  |  |
| Unemployed |  | 20 (10.3%) | 21 (1.9%) |  |  |
| Retired | | 2 (1.0%) | 4 (2.1%) |  |  |
|  | |  |  |  |  |
| PSS-10 score (SD) | | 2.61 (0.73) | 2.89 (0.80) | T-test, t = 3.60 | 0.0003 |

**S2 Table.** Sociodemographics of the Taiwanese Respondents (All Subjects, n=2,727)

| Variables | Distribution |
| --- | --- |
| Age (years) | Min: 18  Max: 100  Mean: 32.85  S.D.: 11.03 |
| Gender | Male 749 (27.5%)  Female 1908 (69.9%)  Other 69 (2.5%) |
| Education | Graduate 878 (32.2%)  College 1625 (59.6%)  Senior High and below 223 (9.2%) |
| Employment | Student 637 (23.4%)  Full-time 1457 (53.6%)  Part-time 136 (5.0%)  Self-employed 196 (7.2%)  Not employed 210 (7.7%)  Retired 82 (3.0%) |

**S3 Table.** Differences in the Coping Strategies among matched Domestic and Overseas Taiwanese Participants (n=386)

| Coping strategies | Domestic (n=193) | Overseas  (n=193) | Diff. (T-test) |
| --- | --- | --- | --- |
| Q1. Information from the government | 4.93 ^a^ | 3.97 | p < 0.001 |
| Q2. Face-to-face interactions with friends and family | 4.30 | 4.02 | p = 0.03 |
| Q3. Phone calls or other long-range interactions with friends and family | 4.54 | 4.56 | p = 0.85 |
| Q4. Face-to-face interactions with colleagues | 3.51 | 3.15 | p = 0.005 |
| Q5. Phone calls or other long-range interactions with colleagues | 3.87 | 4.06 | p = 0.14 |
| Q6. Social media | 4.24 | 4.06 | p = 0.14 |
| Q7. Video games (alone) | 4.17 | 3.61 | p = 0.003 |
| Q8. Video games (online) | 3.76 | 3.42 | p = 0.04 |
| Q9. Watching TV shows or movies | 4.52 | 4.49 | p = 0.82 |
| Q10. Dedicating myself to helping others | 4.26 | 4.08 | p = 0.09 |
| Q11. Dedicating myself to preparing for the crisis | 4.34 | 4.06 | p = 0.01 |
| Q12. Dedicating myself to following the government's advice | 4.70 | 4.05 | p < 0.001 |
| Q13. Dedicating myself to my work/vocation | 4.26 | 4.08 | p = 0.16 |
| Q14. Dedicating myself to an activity or hobby | 4.96 | 4.73 | p = 0.02 |
| Q15. God, religion or spirituality | 2.95 | 2.70 | p = 0.11 |
| Q16. Knowledge of actions taken by the government or civil services | 4.98 | 4.29 | p < 0.001 |

^a^Each item scoring is 1-6: 1- strongly disagree, 6- strongly agree
